# Pituitary tumor transforming gene-1 haplotypes and risk of pituitary adenoma: a case-control study

**DOI:** 10.1186/1471-2350-12-44

**Published:** 2011-03-25

**Authors:** Shuai Chen, Lan Xiao, Zhixiong Liu, Jinfang Liu, Yunsheng Liu

**Affiliations:** 1 Gamma Knife Treatment and Research Center, Xiangya Hospital, Central South University, Changsha, 410008 PR China; 2 Cancer Research Institute, Central South University, Changsha, 410078 PR China

## Abstract

**Background:**

It has been suggested that pituitary adenoma results from accumulation of multiple genetic and/or epigenetic aberrations, which may be identified through association studies. As pituitary tumor transforming gene-1 (*PTTG1*)/securin plays a critical role in promoting genomic instability in pituitary neoplasia, the present study explored the association of *PTTG1 *haplotypes with the risk of pituitary adenoma.

**Methods:**

We genotyped five *PTTG1 *haplotype-tagging SNPs (htSNP) by PCR-RFLP assays in a case-control study, which included 280 Han Chinese patients diagnosed with pituitary adenoma and 280 age-, gender- and geographically matched Han Chinese controls. Haplotypes were reconstructed according to the genotyping data and linkage disequilibrium status of the htSNPs.

**Results:**

No significant differences in allele and genotype frequencies of the htSNPs were observed between pituitary adenoma patients and controls, indicating that none of the individual *PTTG1 *SNPs examined in this study is associated with the risk of pituitary adenoma. In addition, no significant association was detected between the reconstructed *PTTG1 *haplotypes and pituitary adenoma cases or the controls.

**Conclusions:**

Though no significant association was found between *PTTG1 *haplotypes and the risk of pituitary adenoma, this is the first report on the association of individual *PTTG1 *SNPs or *PTTG1 *haplotypes with the risk of pituitary adenoma based on a solid study; it will provide an important reference for future studies on the association between genetic alterations in *PTTG1 *and the risk of pituitary adenoma or other tumors.

## Background

Pituitary tumors are commonly encountered benign monoclonal adenomas that arise from cells of the anterior pituitary gland, accounting for approximately 15% of all diagnosed intracranial tumors [1]. These monoclonal tumors arise from highly differentiated cells expressing hormone gene products, including growth hormone, prolactin, adrenocorticotrophic hormone, thyroid-stimulating hormone, follicle-stimulating hormone and luteinizing hormone. Pituitary adenomas may be functional and actively secrete hormones, leading to characteristic clinical features such as acromegaly, Cushing's disease and hyperprolactinaemia. Commonly, they are non-functional, leading primarily to hypogonadism and compressive pituitary failure [1]. Previous longitudinal incidence surveys reported that the prevalence of pituitary adenoma had been steadily rising over the last decades [2,3]. A latest epidemiological study based on a well-defined population in the UK reveals that the prevalence of pituitary adenoma is 77.6/100,000, about four-fold higher than previously thought [4].

Several genetic and epigenetic alterations have been observed in pituitary tumorigenesis. In addition to classic cancer genes [5,6], there are a significant number of genetic or epigenetic alterations in pituitary tumors that target cell cycle regulators [7]. A completely new cell cycle pathway involved in pituitary oncogenesis is represented by pituitary tumor transforming gene-1 (*PTTG1*)/securin, an oncogenic molecule first identified in GH4 rat pituitary tumor cells [8]. Playing multiple roles in cell cycle regulation at different stages, *PTTG1 *is involved in the mitotic checkpoint that prevents abnormal chromosome segregation [7]. It is overexpressed in a variety of endocrine-related tumors, especially pituitary, thyroid, breast, ovarian, and uterine tumors, as well as nonendocrine-related cancers in the central nervous, pulmonary, and gastrointestinal systems [9]. Increased *PTTG1 *mRNA expression in pituitary tumor tissue has been confirmed in several studies [10,11].

While many studies are focused on exploring the *PTTG1*-mediated tumorigenic mechanisms, none was conducted to study the association of genetic alterations in *PTTG1 *with the risk of pituitary adenoma. In the present study, we are the first to have investigated the association of individual *PTTG1 *SNPs or *PTTG1 *haplotypes with the risk of pituitary adenoma. To comprehensively study the genetic variants of *PTTG1 *associated with susceptibility to pituitary adenomas, we genotyped five *PTTG1 *haplotype-tagging SNPs (htSNP) using PCR-RFLP in a case-control study, which included 280 pairs of age-, gender- and geographically matched Han Chinese people. The five htSNPs, including one in the 5'-flanking region, three in the intronic regions, and one in the 3'-flanking region of the *PTTG1 *gene, appropriately capture all the common haplotype blocks reconstructed in HapMap Phase III data [12].

## Methods

### Specimens

Blood specimens were collected from 280 Han Chinese patients diagnosed with pituitary adenoma at Xiangya Hospital of Central South University between October 2008 and August 2010. None of the patients had received any treatment before blood sampling. As controls, blood samples were collected from 280 age-, gender- and geographically matched Han Chinese individuals without a history of tumor at Xiangya Hospital of Central South University between May and December, 2007. None of the subjects had a family history of pituitary adenoma. None of the pituitary adenoma patients had a history of familial Cushing's syndrome, multiple endocrine neoplasia type 1 (MEN1) or carney complex. Blood specimens were obtained after informed consent from all subjects. This study was approved by the Ethic Committee of Xiangya Hospital.

### Tagging SNP Selection

HapMap SNP Phase III data [12] were used to determine the frequency of SNPs among Han Chinese. Eighty-four SNPs were obtained from a 17.52-kb region of *PTTG1 *from 2 kb upstream of the transcriptional start site to 4 kb downstream of the 3' untranslated region. Tagging SNP selection was done using the Haploview program. The Haploview program implemented a htSNP selection method proposed by Carlson and colleagues, which selects a set of htSNPs such that each SNP considered has *r*^2 ^greater than a prespecified threshold with at least one of the htSNPs [13]. In our selection, only SNPs with minor allele frequency greater than 10% were considered, and the threshold of pairwise linkage disequilibrium (LD) was set as *r*^2 ^= 0.8. A total of five htSNPs were selected among 84 SNPs considered across *PTTG1*, including one in the 5'-flanking region, three in the intronic regions, and one in the 3'-flanking region (Additional file 1).

### Genotyping

Genomic DNA from blood specimens was isolated using standard proteinase K digestion and phenol-chloroform extraction. The five *PTTG1 *htSNPs were amplified by PCR. The sequences of PCR primers are reported in Additional file 2. The PCR reaction was carried out in a total volume of 25 μL, containing 50 to 100 ng of genomic DNA, 1 unit of Premix Taq™ DNA polymerase (Takara, Japan), 0.2 μmol/L of each primer, 1× Ex Taq Buffer (Mg2+ Plus), 0.25 mmol/L of each deoxynucleotide triphosphate. Genotyping for the htSNPs was done by RFLP with restriction endonucleases (Additional file 2). The different alleles were identified on a 4% agarose gel and visualized with ethidium bromide.

### Linkage Disequilibrium (LD) and haplotype Analysis

Pairwise measures of LD measured by Lewontin coefficient (*D'*) and squared correlation coefficient (*r*^2^) between the genotyped SNPs were calculated with the Haploview program [14]. The frequencies of individual haplotypes were estimated from the genotype data using the SHEsis program [15], which implement a Full-Precise-Iteration algorithm for reconstructing haplotypes. Haplotypes with a frequency less than 0.05 were not considered in the analysis. Logistic regression analysis was done using SAS PROC LOGISTIC to estimate the odds ratios (OR) and 95% confidence intervals (95% CI) of individual SNPs or haplotypes.

### Statistical Analysis

Hardy-Weinberg equilibrium analysis for genotype distribution in controls was carried out by a Chi-square goodness-of-fit test. Differences in genotype and allele frequencies between cases and controls were determined using Chi-square test. Logistic regression was performed to assess OR and 95% CI. All the statistical analyses were implemented with SAS 9.1.3. The statistical significance level of this study was set at two-sided α = 0.05.

## Results

As this was an age- and gender-matched cases-control study, there was no significant difference in sex and age between pituitary adenomas patients and controls (Table 1), and therefore adjustment for age and sex was not needed in data analysis.

**Table 1:** Characteristics of Study Samples

	Cases (n = 280), n (%)	Controls (n = 280), n (%)	P*
Age (mean ± SD)	42.7 ± 13.5	42.3 ± 12.4	0.745
Gender			
Male	142 (50.7)	142 (50.7)	1.0
Female	138 (49.3)	138 (49.3)	
Histology			
CA	34 (12.2)		
SA	32 (11.4)		
TA	42 (15)		
LA	84 (30)		
NCA	88 (31.4)		

**Note**: CA, corticotrophic adenoma; SA, somatotrophic adenoma; TA, thyrotrophic adenoma; LA, lactotrophic adenoma (i.e. prolactinoma); NCA, null cell adenoma. *Two-sided Chi-square test was applied to Gender and independent t test was applied to age.

As shown in Table 2, among the five htSNPs, rs2910201 and rs3811999 were found to depart significantly from Hardy-Weinberg equilibrium in controls and therefore excluded from later analyses. No significant difference in allele and genotype frequencies at any of the remaining three polymorphic sites (rs1895320, rs2910200, and rs68827420) was observed between pituitary adenoma patients and controls (Table 3).

**Table 2:** Hardy-Weinberg Equilibrium (HWE) Test on Controls in the Study

Restriction Enzyme	Reference SNP ID (rs)	HWE Test P Value	Chromosome Position	Minor Allele
Tsp45 I	rs1895320	> 0.05	159782164	C
Bsl I	rs2910200	> 0.05	159782569	T
Mbo II	rs2910201	< 0.001	159782950	T
Mnl I	rs3811999	< 0.001	159779450	T
Bsl I	rs6882742	> 0.05	159790342	C

**Table 3:** Genotype and Allelic Frequencies of *PTTG1 *htSNPs among Pituitary Adenoma Cases and Controls and Associations with Risk of Pituitary Adenoma

	Cases/Controls	OR (95% CI)	*P*
rs1895320			
Genotype			
CC	2/4	0.46 (0.08-2.57)	0.68^a^
CT	84/88	0.91 (0.64-1.31)	0.67^b^
TT	194/188	1.00	0.45^c^
Allele			
C	88/96	0.90 (0.66-1.24)	
T	472/464	1.00	0.52*
rs2910200			
Genotype			
TT	4/4	1.12 (0.27-4.58)	0.73^d^
TC	72/54	1.48 (0.99-2.22)	0.06^e^
CC	202/222	1.00	1.00^f^
Allele			
T	80/62	1.35 (0.95-1.93)	
C	476/498	1	0.10*
rs6882742			
Genotype			
CC	4/10	0.36 (0.11-1.16)	0.27^g^
CT	90/104	0.80 (0.56-1.14)	0.26^h^
TT	176/166	1.00	0.11^i^
Allele			
C	98/124	0.78 (0.58-1.05)	
T	442/436	1.00	0.10*

**Note**: As certain genotypes have expected count less than 5 in cases or controls, separate Fisher's exact tests and Chi-square tests are performed where appropriate. a, Fisher's exact P value for rs1895320 CC vs. CT; b, Chi-square P value for rs1895320 CT vs. TT; c, Fisher's exact P value for rs1895320 CC vs. TT; d, Fisher's exact P value for rs2910200 TT vs. TC; e, Chi-square P value for rs2910200 TC vs. CC; f, Fisher's exact P value for rs2910200 TT vs. CC; g, Fisher's exact P value for rs6882742 CC vs. CT; h, Chi-square P value for rs6882742 CT vs. TT; i, Fisher's exact P value for rs6882742 CC vs. TT; *Chi-square P value.

**Figure 1:**
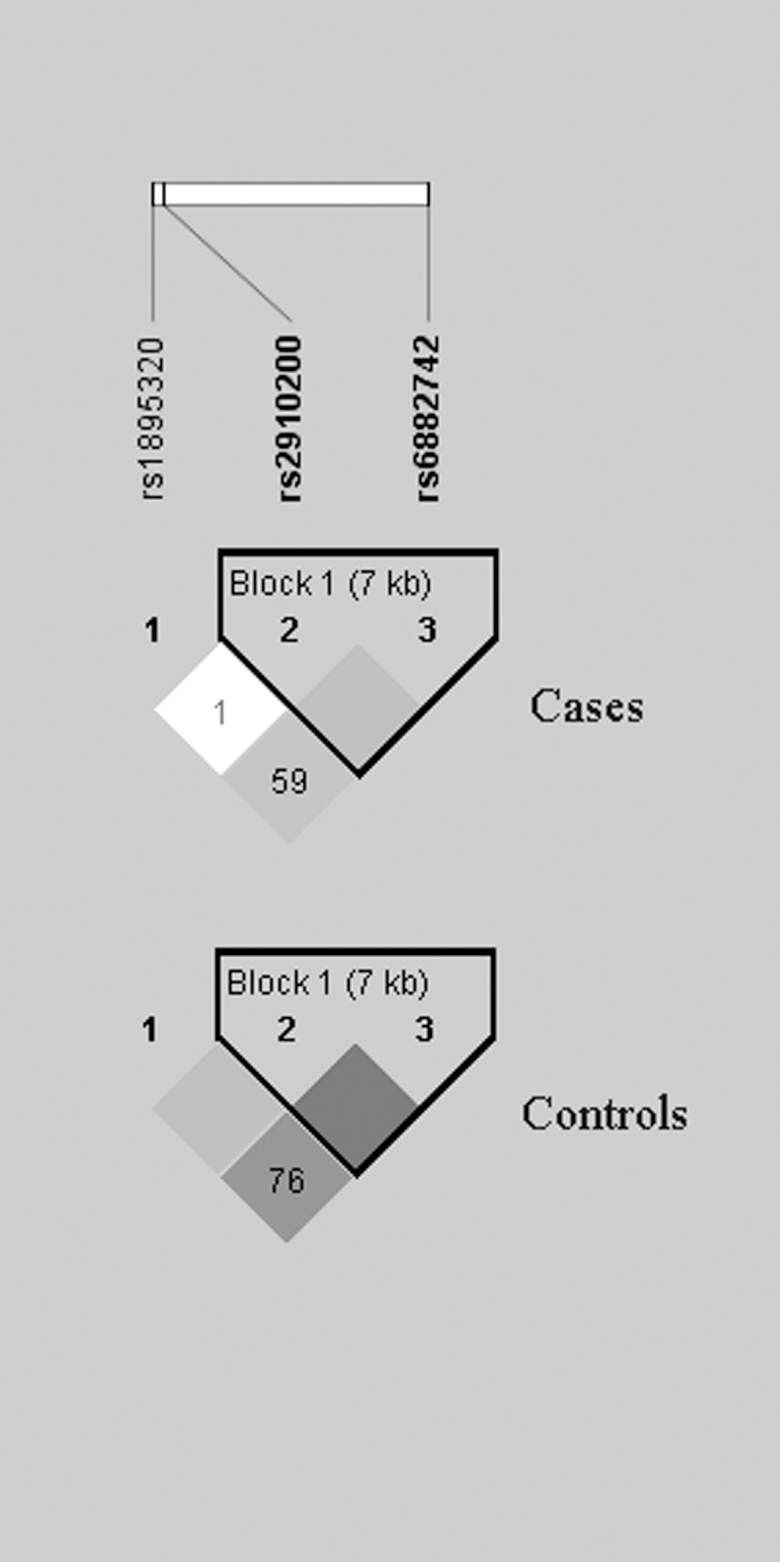
**LD Maps of Three htSNPs in Pituitary Adenoma Cases and Controls**. The Value in each diamond is measured as *D' *corresponding to the dark gray-to-white color gradient. Dark gray diamonds without a number indicate that the value of *D' *was 1.

*D' *value and *r*^2 ^for rs1895320, rs2910200, and rs6882742 were calculated according to the genotyping data reported in Table 3. The different degrees of LD between cases and controls are summarized in Table 4. LD maps measured by *D' *in cases and controls shows rs2910200 and rs6882742 were in LD with each other in both cases and controls (*D' *> 0.8) (Figure 1). Moreover, rs1895320 and rs2910200 had weaker LD in cases (*D' *= 0.012, *r*^2 ^= 0.0) than in controls (*D' *= 1.0, *r*^2 ^= 0.026) (Table 4; Figure 1). According to the genotyping data in pituitary adenoma patients and controls, 2-SNP haplotypes (rs1895320 and rs2910200; rs2910200 and rs68827420) were reconstructed (Table 5). Haplotypes with frequencies greater than 0.05 were subject to further analysis. As shown in Table 5, three 2-SNP haplotypes for rs1895320 and rs2910200 or for rs2910200 and rs6882742 accounted for 100% of the haplotypes in controls, respectively; the frequencies of all the 2-SNP haplotypes were not significantly different between pituitary adenoma patients and controls. In addition, no significant association was found between the 2-SNP haplotypes and histological classification of pituitary adenoma (data not shown).

**Table 4:** D' and r^2 ^between Pairs of Three *PTTG1 *htSNPs in Pituitary Adenoma Cases and Controls

htSNP Pairs	D' Cases/Controls	r^2 ^Cases/Controls
rs1895320 rs2910200	0.012/1.000	0.000/0.026
rs1895320 rs6882742	0.595/0.764	0.303/0.425
rs2910200 rs6882742	0.999/1.000	0.038/0.035

**Note**: Values of *D' *and *r^2 ^*were calculated with the Haploview program.

**Table 5:** Frequencies of Estimated 2-SNP Haplotypes of *PTTG1 *in Pituitary Adenoma Cases and Controls

	rs1895320 -- rs2910200 2-SNP Haplotype			rs2910200 -- rs6882742 2-SNP Haplotype		
	CC	TT	TC	CC	TT	CT
						
Cases (%)/Controls (%)	13.4/17.1	12.0/11.1	72.2/71.8	18.2/22.1	14.5/11.1	67.2/66.8
OR (95% CI)*	0.77 (0.56-1.07)	1.13 (0.78-1.63)	1.12 (0.86-1.46)	0.78 (0.58-1.06)	1.36 (0.95-1.95)	1.02 (0.79-1.32)
*P*	0.120	0.532	0.416	0.107	0.089	0.865

**Note**: Haplotypes with frequencies > 5% were included. *Calculated with the SHEsis program.

## Discussion

Pituitary tumors are common intracranial neoplasms that cause significant morbidity through mass effects and/or inappropriate secretion of pituitary hormones [16]. The prevalence of pituitary adenoma has dramatically increased in the past decade [4].

*PTTG1/*securin is a vital component of the spindle checkpoint controlling faithful chromatid separation. It inhibits separase, the major effector for chromosome segregation during mitosis [17]. Overexpression of *PTTG1 *causes cell transformation and induces aneuploidy [18,19], whereas either abundance or loss of it can lead to dysregulated G2/M checkpoint surveillance, resulting in abnormal mitosis and chromosomal instability. Absence of this gene results in a decrease in the incidence of pituitary tumors in pRB heterozygous mice, probably by triggering ARF/p53/p21-dependent senescence [20,21]. In contrast, overexpression *PTTG1 *in the pituitary in transgenic mice leads to pituitary hyperplasia and focal microadenomas [22]. All the previous findings indicate that *PTTG1 *is required for pituitary tumorigenesis, which warrant our study to explore possible association of genetic alterations in *PTTG1 *with the risk of pituitary adenoma, using htSNP and haplotype analyses to comprehensively capture various genetic variants of *PTTG1 *in a Han Chinese population. However, our results revealed no significant association between individual *PTTG1 *htSNPs or reconstructed *PTTG1 *haplotypes and the risk of pituitary adenoma, suggesting that genetic variation in *PTTG1 *is not an imporant contributing factor to pituitary tumorigenesis. *PTTG1 *seems to promote pituitary tumor formation through a variety of other ways.

Zhang et al. reported that *PTTG1 *mRNA was overexpressed in more than 90% of all types of pituitary tumors [10]. Based on our results, it seems that the *PTTG1 *mRNA overexpression in pituitary adenoma is more likely through epigenetic mechanisms rather than variation in the DNA sequence of *PTTG1*. Holt et al. reported that PTTG1 was regulated by cyclin-dependent kinase (CDK)-mediated phosphorylation [23], suggesting a link between the control of the cell cycle by CDKs and PTTG1. Moreover, PTTG1 activates β-fibroblast growth factor, c-myc and cyclin D3 to enhance cell proliferation [24-26], and interacts with Ku and p53 to participate in DNA damage/repair and apoptosis [27]. Interplay between PTTG1 and the cell signaling molecules, rather than DNA sequence variation in the *PTTG1 *gene, may actually play an important role in pituitary tumorigenesis. Nevertheless, individual SNP analysis and/or haplotype analysis on genes of the interaction partners of PTTG1, or on newly identified candidate genes involved in pathogenesis of pituitary adenoma, such as the bone morphogenetic protein-4 (BMP-4) gene and the RWD-containing sumoylation enhancer (RSUME) gene [28], may yield interesting results.

## Conclusions

Though no significant association was found between *PTTG1 *haplotypes and the risk of pituitary adenoma, this is the first report on the association of individual *PTTG1 *SNPs or *PTTG1 *haplotypes with the risk of pituitary adenoma based on a solid study; it will provide an important reference for future studies on the association between genetic alterations in *PTTG1 *and the risk of pituitary adenoma or other tumors.

## Competing interests

The authors declare that they have no competing interests.

## Authors' Contributions

SC participated in study design, collected data, carried out data analysis, and drafted the manuscript. LX participated in study design, carried out data analysis, and performed data check and proofreading. ZL participated in data collection, carried out data analysis, and performed data check and proofreading. JL participated in study design, data collection and data analysis. YL participated in study design and data analysis, drafted the manuscript, and performed data check and proofreading. All authors have read and approved the final manuscript.

## Pre-publication history

The pre-publication history for this paper can be accessed here:

http://www.biomedcentral.com/1471-2350/12/44/prepub

## Supplementary Material

Additional file 1 **Haplotype Tagging SNPs of the *PTTG1 *Gene in the Chinese Han Population**. SNP position and minor allele frequency are based on HapMap SNP Phase III data http://hapmap.ncbi.nlm.nih.gov/cgi-perl/gbrowse/hapmap28_B36. *Genotyped in reverse direction as opposed to A/G in forward direction. Click here for file

Additonal file 2 **Primers and Restriction Endonucleases Used for *PTTG1 *Genotyping**. *FP, Forward primer; RP, Reverse primer; ‡ The polymorphic alleles are identified following cleavage by restriction endonucleases, which yield fragments of different sizes for different alleles. Click here for file
