# Supplementary material for: Pituitary tumor transforming gene-1 haplotypes and risk of pituitary adenoma: a case-control study
Source: BMC Med Genet. 2011 Mar 25;12:44. doi: 10.1186/1471-2350-12-44 (PMC3078851; doi:10.1186/1471-2350-12-44)
Supplement: Additional file 1 — Haplotype Tagging SNPs of the PTTG1 Gene in the Chinese Han Population. SNP position and minor allele frequency are based on HapMap SNP Phase III data http://hapmap.ncbi.nlm.nih.gov/cgi-perl/gbrowse/hapmap28_B36. *Genotyped in reverse direction as opposed to A/G in forward direction. [file 1471-2350-12-44-S1.DOC]

**Additional file 1.** Haplotype Tagging SNPs of the *PTTG1* Gene in the Chinese Han Population

| Reference SNP ID (rs) | Chromosome Position | Location in Gene | Alleles | Minor Allele (Frequency) |
| --- | --- | --- | --- | --- |
| rs1895320 | 159782164 | intron 2 | T/C* | C (0.161) |
| rs2910200 | 159782569 | intron 3 | C/T | T (0.157) |
| rs2910201 | 159782950 | intron 3 | C/T | T (0.318) |
| rs3811999 | 159779450 | 5`-flanking | C/T | T (0.163) |
| rs6882742 | 159790342 | 3`-flanking | T/C | C (0.208) |

**Note**: SNP position and minor allele frequency are based on HapMap SNP Phase III data (<http://hapmap.ncbi.nlm.nih.gov/cgi-perl/gbrowse/hapmap28_B36>). *Genotyped in reverse direction as opposed to A/G in forward direction.
