# Supplementary material for: Pituitary tumor transforming gene-1 haplotypes and risk of pituitary adenoma: a case-control study
Source: BMC Med Genet. 2011 Mar 25;12:44. doi: 10.1186/1471-2350-12-44 (PMC3078851; doi:10.1186/1471-2350-12-44)
Supplement: Additonal file 2 — Primers and Restriction Endonucleases Used for PTTG1 Genotyping. *FP, Forward primer; RP, Reverse primer; ‡ The polymorphic alleles are identified following cleavage by restriction endonucleases, which yield fragments of different sizes for different alleles. [file 1471-2350-12-44-S2.DOC]

**Additional file 2.** Primers and Restriction Endonucleases Used for *PTTG1* Genotyping

Primer Restriction endonuclease † Product

Length (bp)Restriction EndonucleaseIncubation

Temperature (°C)Specific

Allele (Position) ‡htSNP IDPrimer sequences *rs1895320 FP: TTTGGTAGTGGCATGGCTGTG 149Tsp45 I65C (131) RP: TGGAGCTGGACGAGACATTT rs2910200 FP: GCCAAAAAGGTAAGTGTTGG 127Bsl I55C (106) RP: TAACCTCTATTTCCCTTCCACrs2910201FP: TTTTGCCTCACCTGTCCTTC330Mbo II37C (236) RP: TCCACATGCTAATGTCATCTT Rs3811999 FP: TAACGTGTATCCTTTTTGAATG 200Mnl I37C (139) RP:CTCTATTCACTTTCACGCTAGTCTC rs6882742 FP: GGTTGTTGCTATTTTGTTCTGAC 119Bsl I55C (99) RP: GTATCCTGAGAGGCAGGCCCA

**Note**: *FP, Forward primer; RP, Reverse primer; ‡ The polymorphic alleles are identified following cleavage by restriction endonucleases, which yield fragments of different sizes for different alleles.
